# Tumor evolution and intratumor heterogeneity of an epithelial ovarian cancer investigated using next-generation sequencing

**DOI:** 10.1186/s12885-015-1077-4

**Published:** 2015-02-26

**Authors:** Jung-Yun Lee, Jung-Ki Yoon, Boyun Kim, Soochi Kim, Min A Kim, Hyeonseob Lim, Duhee Bang, Yong-Sang Song

**Affiliations:** 1 Department of Obstetrics and Gynecology, Seoul National University, College of Medicine, 101 Daehak-ro, Jongno-gu, Seoul, 110-744 South Korea; 2 College of Medicine, Seoul National University, Seoul, 110-744 Republic of Korea; 3 Cancer Research Institute, Seoul National University College of Medicine, Seoul, 110-799 Republic of Korea; 4 Department of Pathology, Seoul National University College of Medicine, Seoul, 110-744 Republic of Korea; 5 Department of Chemistry, Yonsei University, Room 437, Science Building, 50 Yonsei-ro, Seodaemun-gu, Seoul, 120-749 South Korea; 6 Major in Biomodulation, World Class University, Seoul National University, Seoul, 151-742 Republic of Korea

**Keywords:** High grade serous ovarian cancer, Whole exome sequencing, Intratumor heterogeneity, Peritoneal seeding, Transcoelomic metastasis

## Abstract

**Background:**

The extent to which metastatic tumors further evolve by accumulating additional mutations is unclear and has yet to be addressed extensively using next-generation sequencing of high-grade serous ovarian cancer.

**Methods:**

Eleven spatially separated tumor samples from the primary tumor and associated metastatic sites and two normal samples were obtained from a Stage IIIC ovarian cancer patient during cytoreductive surgery prior to chemotherapy. Whole exome sequencing and copy number analysis were performed. Omental exomes were sequenced with a high depth of coverage to thoroughly explore the variants in metastatic lesions. Somatic mutations were further validated by ultra-deep targeted sequencing to sort out false positives and false negatives. Based on the somatic mutations and copy number variation profiles, a phylogenetic tree was generated to explore the evolutionary relationship among tumor samples.

**Results:**

Only 6% of the somatic mutations were present in every sample of a given case with *TP53* as the only known mutant gene consistently present in all samples. Two non-spatial clusters of primary tumors (cluster P1 and P2), and a cluster of metastatic regions (cluster M) were identified. The patterns of mutations indicate that cluster P1 and P2 diverged in the early phase of tumorigenesis, and that metastatic cluster M originated from the common ancestral clone of cluster P1 with few somatic mutations and copy number variations.

**Conclusions:**

Although a high level of intratumor heterogeneity was evident in high-grade serous ovarian cancer, our results suggest that transcoelomic metastasis arises with little accumulation of somatic mutations and copy number alterations in this patient.

**Electronic supplementary material:**

The online version of this article (doi:10.1186/s12885-015-1077-4) contains supplementary material, which is available to authorized users.

## Background

Epithelial ovarian cancer is the fifth leading cause of cancer death among women in the USA [1]. The major reason for the poor prognosis is the fact that more than 75% of patients are diagnosed with advanced stage disease characterized by metastasis to the peritoneal cavity. The metastatic patterns of ovarian cancer differ from those of most other malignant epithelial disease. Transcoelomic is the most common route of metastasis in epithelial ovarian cancer and contributes to the significant morbidity and mortality associated with this cancer [2]. Given the high recurrence rate and poor long-term survival of women with advanced stage disease, there is a strong need to document the unique metastatic patterns of epithelial ovarian cancer by comparing the differences in genetic profiles between primary and metastatic lesions.

With the recent development of next-generation sequencing (NGS) technology, the Cancer Genome Atlas (TCGA) researchers have identified molecular abnormalities related to the pathophysiology, clinical outcome, and potential therapeutic targets in high-grade serous ovarian cancer (HGSC) [3]. The TCGA study provides a large-scale integrative view of the aberration in HGSC with extensive heterogeneity between individual tumors. However, it is not certain whether the genomic alterations found in single tumor biopsy samples from primary tumors are maintained in metastatic lesions. Furthermore, intratumor heterogeneity has been proposed as the main cause of treatment failure and drug resistance in ovarian cancer and other primary cancers [4]. Recently, NGS technology has led to progress in the evaluation of intratumor heterogeneity in various cancers [5-8]. In the field of HGSC, intratumor heterogeneity has been evaluated within primary tumors and associated metastatic sites, and the divergence of genetic variants was observed [5]. Despite evident intratumor heterogeneity within individual patients, little is known about how metastatic tumors further evolve compared to primary sites. The aim of this study was to explore the mutational profiles of primary tumors and associated metastatic lesions, and to identify the evolutionary relationship between primary and metastatic clones with NGS technology.

## Methods

### Patient information and sample preparation

**Figure 1:**
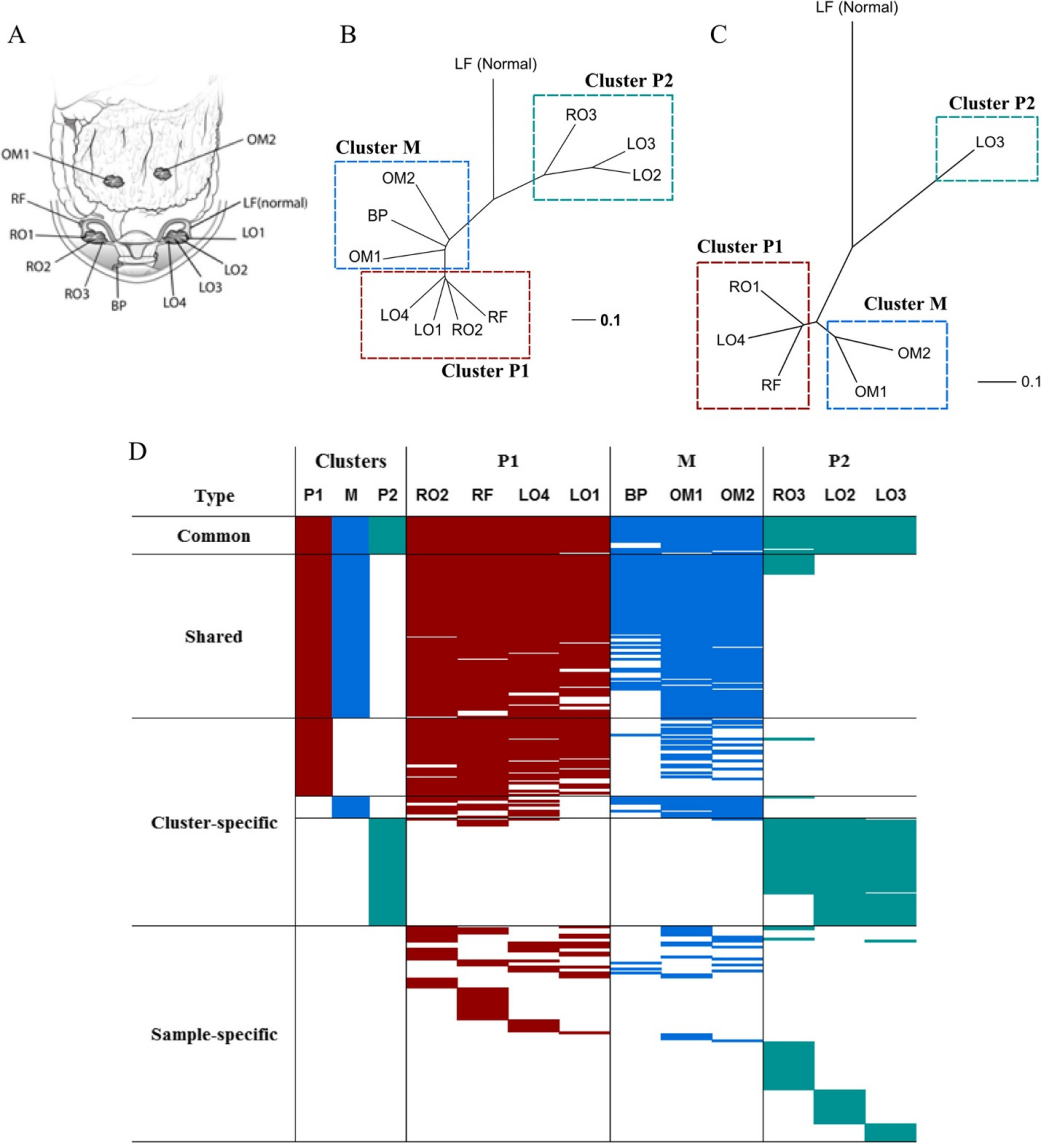
**Intra-tumoral mutational profiles of HGSC. (A)** Sampling sites of tumor and normal control tissues. **(B)** Phylogenetic tree of somatic mutations. **(C)** Phylogenetic tree of somatic copy number variations. **(D)** Patterns of somatic mutations across samples. HGSC: high grade serous ovarian cancer, RO: right ovary, RF: right fimbriae, LO: left ovary, LF: left fimbriae, BP: bladder peritoneum, OM: omentum.

A 71-year-old female was diagnosed with stage IIIC ovarian cancer at the time of sample collection. She had no family history for breast or ovarian cancer. She underwent BRCA1/2 germline mutation testing (Integrated BRACAnalysis®) and no mutation was found. Preoperative CA-125 level was 336 U/mL. She underwent cytoreductive surgery followed by platinum-based chemotherapy. During cytoreductive surgery, a right ovarian cystic mass of 10 × 9 × 8 cm in size was found. Multiple solid lesions were found inside the right ovarian cystic mass. A left ovarian tumor measuring 6 × 5 × 4 cm and consisting mostly of solid tissue was also found. Seven samples were taken randomly from the solid portions of both ovaries with a certain distance retained between each sample. All tissues consisted of >70% high-grade (FIGO grade 3) serous adenocarcinoma cells based on pathological review. Adjacent normal tissues from the left fimbriae and blood were also collected to serve as normal controls. Eleven tumor samples were collected from the ovaries, right fimbriae, bladder peritoneum, and omental lesions during surgery under the supervision of our pathologist (Min A Kim) (Figure 1A).

This patient had no evidence of recurrence at the time of publication and 12 months had passed since the completion of first-line treatment. This was a platinum-sensitive case (>6 months after first-line treatment completion). This study was approved by the Institutional Review Board (IRB) at Seoul National University Hospital (Registration number: C-1305-546-487) and performed in compliance with the Helsinki Declaration. We obtained informed consent for samples to be used in research. Written informed consent was obtained from the patient for publication of the case report including any accompanying images and disclosure of sequence data.

### Library construction, exome capture, and sequencing

Genomic DNA was extracted separately from each sample (Qiagen, Valencia, CA, USA) and shotgun libraries were constructed by shearing 3 μg of genomic DNA. The SureSelect Human All Exon V4 + UTRs kit (Agilent, Santa Clara, CA, USA) was used to capture 71 Mbps of exons and UTRs, according to the manufacturer’s protocol, which were subsequently sequenced on an Illumina HiSeq2500 (Additional file 1: Tables S1 and S2). Sequencing data are accessible at Sequence Read Archive (SRA, accession number SRS823287).

### Analysis of whole exome sequencing data

Short reads were aligned to the reference human genome (hg19) using Novoalign V2.07.18 with the default options. PCR and optical duplicates were removed using Picard v1.67 MarkDuplicates. Local realignment around the known indels in dbSNP135 and base quality score recalibration were performed using the Genome Analysis Toolkit (GATK) v2.6-4 [9]. Somatic mutations were identified by muTect 1.1.4 with the default options [10], and manually inspected by using Integrative Genome Viewer (IGV) [11]. The variants were annotated using the SeattleSeq Annotator, and then the variants listed in dbSNP132 and in repetitive regions were removed (repeatMasker, tandemRepeat column in SeattleSeq). Intronic, intergenic, near-gene, and synonymous mutations were also excluded. The germline mutations were identified by the GATK Unified Genotyper with the blood sample. Small indels were detected by Dindel v1.01 [12]. To avoid false positive somatic indels, only indels validated manually by IGV and confirmed by multiplex PCR were considered real variants. Candidate driver mutations and functional germline mutations were called based on the results from seven functional prediction algorithms and three conservation score algorithms using ANNOVAR [13] and dbNSFP v2.0 [14] (Additional file 2). All URLs for the analysis programs are listed in Additional file 2.

### Somatic copy number alteration (SCNA) analysis

Genomic DNA (~600 ng) from each sample was processed with SNP chip analysis using the Genome-wide Human SNP Array 6.0 (Affymetrix, Santa Clara, CA, USA) according to the manufacturer’s instructions (Additional file 1: Table S1). Raw data were processed with the Affymetrix SNP6 Copy Number Inference Pipeline developed by Broad Institute using GenePattern modules [15]. Briefly, raw data was calibrated to signal intensities, called genotypes, and then the signal intensities were converted to copy number calls. After refinement of the copy numbers, somatic copy number alterations (SCNA) were called by subtracting the signals in the tumor sample from those in the normal sample. The segments of the SCNA were identified by circular binary segmentation.

For omental samples, whole exome sequencing data was used to detect SCNA. Pair-end read data was processed by the Varscan2 copynumber and copyCaller [16] with whole exome sequencing data of blood and the following non-default parameters: max-segment-size, 250; data-ratio 0.301 for OM1 and 0.306 for OM2. These raw segment data were smoothed and segmented using the ‘DNAcopy’ R package [17] with alpha = 0.01, nperm = 10,000, and trim = 0.025, then the segment values were magnified three times. All SCNA were visualized using Circos plot v0.64 [18].

### Validation of somatic mutations and indels

Since quality control for false negatives is crucial for exploring intratumor heterogeneity, we selected 122 loci primers for multiplex PCR with HiSeq2500 for validation. Primer pairs were designed and synthesized based on column-based methods, pooled, and multiplex PCR was performed with 50 ng of genomic DNA from each sample (Celemics, Seoul, Korea). Subsequently, each product was indexed, mixed, and deeply sequenced on HiSeq2500. Raw data was deindexed and mapped to the reference human genome (hg19) using NovoAlign. Mutascope was used to call somatic mutations, and compared to the whole exome sequencing data [19]. Only the loci with at least 500 reads of both normal and tumor tissue and >5% allelic fraction were used for validation.

### Phylogenetic tree construction and variant classification

A phylogenetic tree was generated to assess the tumor evolutionary patterns in terms of somatic mutations. The phylogenetic analysis followed the method described in a previous report [5]. All point mutations were converted to binary data (0 = no mutation, 1 = somatic mutation) for each sample, and a matrix with sample names in rows and loci in columns was generated. Next, we calculated Pearson correlation coefficients (ρ_xy_) between samples x and y, and 1-ρ_xy_ was considered the distance between x and y. The Neighbor-Joining method [20] and the Unweighted Pair Group Method with Arithmetic Mean (UPGMA) method were applied to cluster samples and construct the phylogenetic tree. We used the ‘ape’ R package [21] for these analyses.

Samples were segregated by cluster P1, cluster P2, and cluster M for further analysis (Figure 1). If any somatic mutation was found in at least three samples in ‘cluster P1’ or at least two samples in ‘cluster P2’ and ‘cluster M’, we concluded that the mutation truly existed in that respective cluster. A mutation was classified as “Common” when it was found in all clusters, as “Shared” when found in any two clusters, as “Cluster-specific” when found in only one cluster, otherwise as “Sample-specific”.

Similar to the somatic mutation analysis, somatic copy number alterations were also converted to weights as follows; δmax [log_10_ L, 1], where L is the segment length, δ = 1 if the segment was amplified, −1 if deleted, or 0 otherwise. A matrix with sample names in rows and altered regions in columns were constructed. Pearson coefficients were calculated, and a phylogenetic tree was generated as described above.

The segments were classified as cluster P1, cluster P2, and cluster M as well. Initially, the cut-off values (log_2_ ratio) for amplified and deleted segments were set to 0.2 and −0.2, respectively. We decided that the segment was altered, either amplified or deleted, only if all samples in each cluster were amplified or all samples were deleted. If any sample in a cluster was altered differently, the segment was neglected. We classified the segments as “Common” when all three clusters had the same sample variation, “Shared” when any two clusters had the same variation, and “Specific” when variation was found in only one cluster. Coding genes (RefSeq database) within each segment were collected and functional analysis was performed using the DAVID functional annotation tool [22] and the GO_BP (Gene Ontology, Biological Process) and KEGG pathway databases.

## Results

Whole coding exons and untranslated regions (71 Mbp) in genomic DNA from seven ovarian tumor sites, three metastatic lesions, and two normal control samples (including a blood sample) were sequenced (Figure 1A). The mean coverage was 92× for tumor tissue and 65× for normal tissue. We sequenced more deeply on two omental tumor samples (211×, 199×) to thoroughly explore the variants in metastatic lesions (Additional file 1: Table S2). A total of 2,248 somatic mutations (3.2/Mb for each sample on average) were identified, and the average number of non-synonymous or splicing site mutations was 122 per sample (range: 77–167) (Additional file 1: Table S3). To avoid overestimation of intratumor heterogeneity, we randomly selected 122 somatic mutations (Additional file 1: Table S4) and performed multiplex PCR followed by ultra-deep re-sequencing (median coverage: 9,647×) for eight samples (Additional file 1: Table S1). The precision, false negative rate, and false positive rate of mutation calling in whole exome sequencing were 93%, 6%, and 1%, respectively. We found no pathologic *BRCA1* and *BRCA2* germline mutation in this patient. Other germline mutations are listed in Additional file 1: Table S5.

Phylogenetic trees were generated with somatic mutation data on 634 loci that were found at least once in the tumor samples. The samples from primary sites were segregated into two clusters (clusters P1 and P2), and the samples from metastatic lesions formed cluster M (Figure 1B). Based on the evolutionary tree, clusters P1 and P2 diverged earlier than cluster P1 and M. Interestingly, clusters P1 and P2 were not united according to the spatial position of sampling sites. These patterns were also observed in the phylogenetic tree based on copy number variations (Figure 1C).

Next, we classified 313 non-synonymous or splicing site mutations into four groups: Common, Shared, Cluster-specific, and Sample-specific (Figure 1D). Only 19 mutations (6%) were found in most samples, the Common group, which showed higher intratumor heterogeneity than previous studies across various cancers [5-8]. Ten non-synonymous mutations in genes including *TP53*, *KIF13A*, and *SPIC* were identified (Table 1), indicating that those mutations were acquired in the early stage of tumorigenesis. Eighty-two (26%) somatic mutations were in the Shared group. All mutations in the Shared group were discovered in both cluster P1 and cluster M, supporting a common evolutionary origin. Also, 25 nonsynonymous mutations were considered as the candidate driver mutations. *TP53* Y220C and *SPIC* E152K in the Common group are only mutations listed in COSMIC database (Table 1). We could not identify any anti-neoplastic therapeutic agents that interact with candidate driver mutations except *PRKCQ* C281S, which was found to interact with sophoretin [23]. However, this mutation is only detected in Cluster P2.

**Table 1:** **Candidate driver mutations affecting characteristics of ovarian cancer**

Type	Genomic position (hg19)	Base change	Gene	Amino acid change	Predicted as damaging^†^	COSMIC (ID)
Common	chr17:7578190	T>C	*TP53*	Y220C	7/7	COSM99720
	chr6:17781485	C>T	*KIF13A*	G1198S	6/7	-
	chr14:25044511	G>A	*CTSG*	R55*	N/A	-
	chr15:92459643	T>C	*SLCO3A1*	Y201H	6/7	-
	chr12:101880256	G>A	*SPIC*	E152K	2/7	COSM458288
Shared (P1, M)	chr10:28905209	T>G	*WAC*	L555*	N/A	-
	chr14:24879362	G>T	*NYNRIN*	G788*	N/A	-
	chr21:37710167	G>T	*MORC3*	G128V	6/7	-
	chr1:154184966	C>G	*C1orf43*	D159H	6/7	-
	chr4:154626187	A>G	*TLR2*	S710G	6/7	-
	chr12:52183202	G>C	*SCN8A*	K1432N	6/7	-
	chr14:23886761	G>C	*MYH7*	S1435C	6/6	-
	chr1:11594572	G>A	*PTCHD2*	W1170*	N/A	-
	chr12:20799431	C>A	*PDE3A*	N753K	6/7	-
	chr2:196545035	G>T	*SLC39A10*	G90V	6/7	-
Cluster P1-specific	chr1:43317062	T>G	*ZNF691*	C176G	6/7	-
	chr13:113893782	G>T	*CUL4A*	D318Y	6/7	-
	chr6:117892084	C>A	*GOPC*	G276V	6/7	-
	chr11:134090616	C>A	*NCAPD3*	W23C	6/7	-
Cluster M- specific	chr1:206944347	T>C	*IL10*	M95V	5/7	-
	chr2:242690697	C>G	*D2HGDH*	S345C	7/7	-
Cluster P2-specific	chr15:80866542	C>G	*ARNT2*	S457*	N/A	-
	chr8:72211468	C>G	*EYA1*	D214H	7/7	-
	chr3:101371645	T>G	*ZBTB11*	H816P	7/7	-
	chr10:6528056	A>T	*PRKCQ*	C281S	7/7	-

^†^Predicted as damaging = (the number of algorithms predicting a damaging mutation)/(the number of available algorithms). The prediction algorithms and their cutoffs were described in the Methods.

*Stop codon.

N/A = not available.

Only 11 somatic mutations were identified in the Cluster-specific group in cluster M, much fewer than those in clusters P1 and P2 (39 and 54, respectively). The mutations classified in cluster M-specific group were dominantly found in most samples of cluster M but not in other clusters. However, all 11 cluster M-specific mutations were also found in at least one sample from cluster P1. In contrast, most cluster P2-specific mutations were found only in cluster P2 (Figure 1D). False negative calling of cluster M-specific mutations was less likely, since the omental samples were deeply exome-sequenced and further validated by multiplex PCR followed by deep re-sequencing. The false negative rate of mutation calling in omental samples calculated with validation sequencing was less than 10%. Therefore, it seems that cluster M diverged from the common ancestry clone of cluster P1 with few additional somatic mutations.

To identify the branching mutation related to the origin of cluster P2, we focused on a subset of cluster P2-specific mutations found in non-cluster P2 samples (Additional file 1: Table S6). The allele frequency of each sample determined by ultra-deep re-sequencing was normalized to the mean allele frequency. The normalized allele frequencies were comparable between cluster P2 and non-cluster P2 samples, but that of *ARNT2* S457* in cluster P2 was about ten-fold higher than in the right fimbriae. This finding supports the notion that the mutation was obtained upon the divergence of cluster P2.

**Figure 2:**
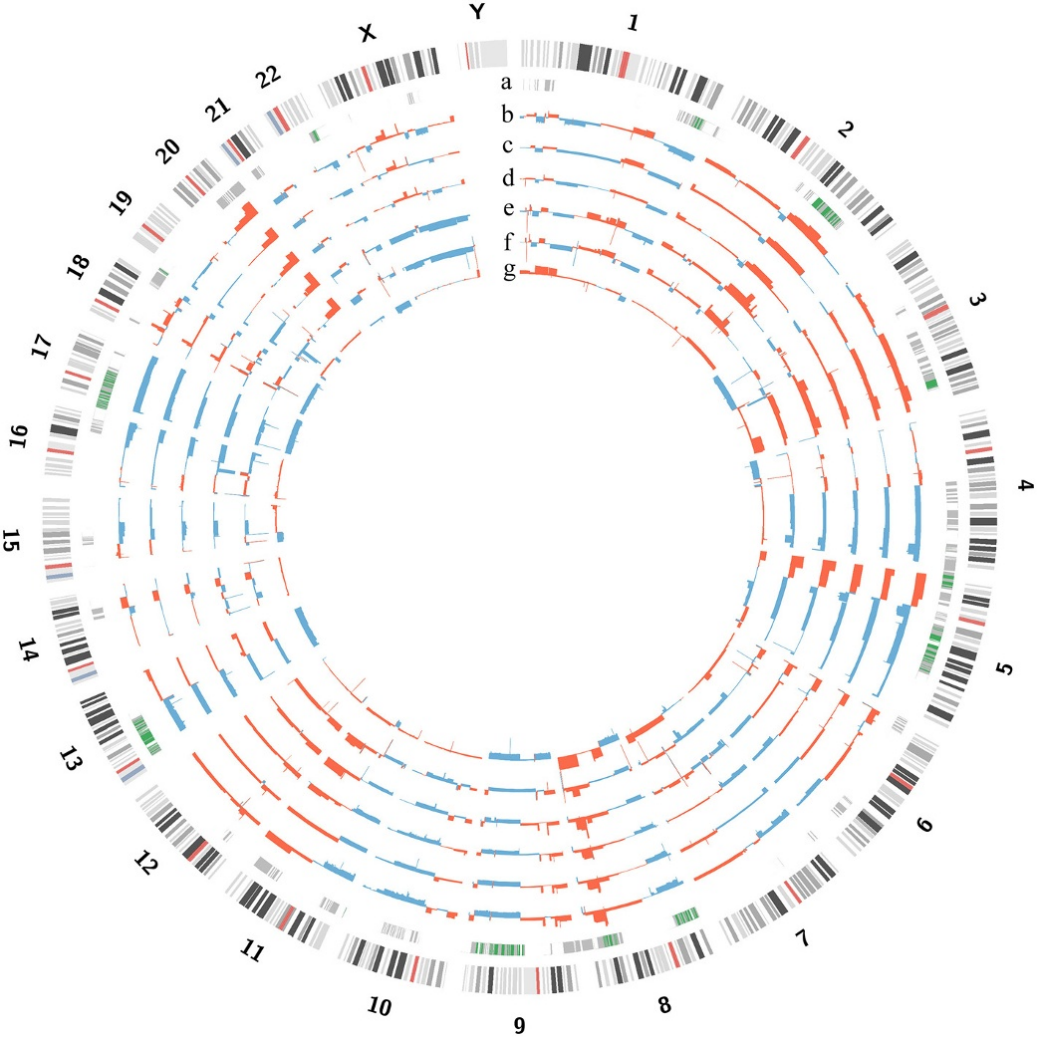
**Genomic profiles of somatic copy number alterations (SCNA). (a)** Common segments (green) and Shared segments (grey). **(b, c, d)** Cluster P1 samples, RO1, RF, and LO4. **(e, f)** Cluster M samples, OM1, and OM2. **(g)** Cluster P2 sample, LO3. Overall the pattern of SCNA in cluster M was similar to the pattern in cluster P1 except for a large deletion on chromosome X. Cluster P2 showed distinct SCNA patterns compared to other clusters. red: amplification, blue: deletion.

SCNA were derived from six tumor samples and a normal sample. The analysis showed that the genomic architectures of samples in cluster M were similar to the patterns in cluster P1, but an arm-scale deletion on chromosome X was observed in cluster M (Figure 2). In contrast, the copy number patterns of cluster P2 were quite different from those of cluster P1 or M, supporting the conclusion that clones in cluster P2 diverged earlier than cluster M. Similar to the somatic mutation classification, we classified “Common segments” when the amplified/deleted segments were observed in all samples, suggesting that the segments formed in the early phase of tumorigenesis. Forty-four Common segments spanning 101 Mb were amplified, and 168 Common segments spanning 245 Mb were deleted. These segments covered 354 genes and 1,835 genes, respectively. Then, we characterized functional pathways affected by these genes. The genes related to ‘skeletal system development’ were enriched in amplified Common segments, and those related to ‘embryonic development ending in birth or egg hatching’ and ‘chemokine signaling pathway’ were enriched in deleted Common segments (Additional file 1: Table S7). The segments were considered a “Shared segment” when amplification or deletion was found in samples from any two clusters. We determined that 154 Shared segments (227 Mb) were amplified and 248 Shared segments (287 Mb) were deleted in both clusters P1 and M. Pathways previously reported to be altered in ovarian cancer, such as the JAK/STAT signaling pathway and the Cytokine-Cytokine Receptor pathway [24], were also identified in these Shared segments, but not in the Common segments. Interestingly, the genes related to blood vessel morphogenesis (31 genes, Benjamin-Hochberg (BH) score 0.094) were deleted Shared segments between cluster P1 and M, but not cluster P2. In contrast, the genes related to hemophilic cell adhesion (50 genes, BH score 9.9x10^−16^) were enriched in the amplified segments found only in cluster P2.

**Figure 3:**
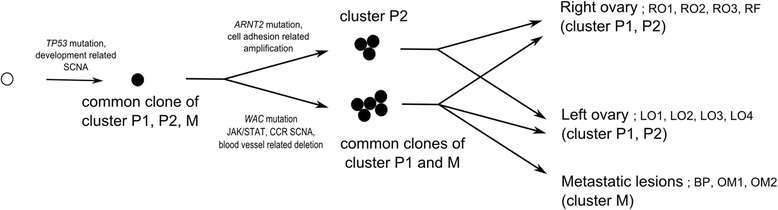
**Evolutionary model of non-spatial clustered metastatic ovarian cancer.** (CCR: cytokine-cytokine receptor pathway, SCNA: somatic copy number alteration).

Phylogenetic tree analysis based on somatic mutation and copy number variation was used to study the clonal relationship between different regions of primary and metastatic tumors (Figure 3). The findings indicate that metastatic lesions derive from a common, ancestral clone within the primary tumor. In cases of bilateral ovarian tumors like the one assessed here, metastatic potential may be gained in the early stages of tumorigenesis.

Based on the SCNA data, we focused on the frequently detected focal SCNA reported in the TCGA data (Additional file 1: Table S8) [3]. Among the top 20 most frequently observed focal amplifications, 12 segments were altered in our study, and only MECOM, TERT, and MYC segments were found among Common amplified regions. Although regions containing KRAS, ID4, MYCL1, and SOX17 were observed as tightly localized amplification peaks, these peaks were observed only clusters P1 and P2 for the patient in our study. Also, among the top 20 most frequent focal deletions, we found that 15% (3 of 20) of focal deletions including RB1 and PPP2R2A were commonly observed in our patient. NF1 is one of the genes shown to be related to intratumor heterogeneity in a previous study [5]. However, NF1 deletions were observed in both clusters in our study. This finding suggests that intratumor heterogeneity might appear differently in each patient. Lastly, we annotated the copy number variation patterns of 22 drug targets listed in the TCGA project for this patient [3]. Although 15 targeted genes were altered in this patient, only 40% (6 of 15) of the targeted genes were altered in all clusters (Additional file 1: Table S9).

## Discussion

Using NGS technology followed by confirmative validation, we were able to identify the clonal evolution of multiple samples collected from both ovaries and metastatic lesions in a single patient. Even though only 6% of somatic mutations were present in all samples, the vast majority of somatic variants found in the metastatic samples were present in the primary tumor samples. All 11 cluster M-specific mutations were found in at least one sample in cluster P1, and no somatic mutation was further accumulated in cluster M. In addition, SCNA showed that the genomic architecture of samples in cluster M were similar to the patterns in cluster P1. These findings suggest that peritoneal seeding arises with little accumulation of somatic mutations and copy number alterations in this patient. We also observed that non-spatial clusters of the primary ovarian cancer samples (cluster P1 and P2) shared a small number of genetic variations (Common mutations and segments), which indicates that metastatic potential developed at an early stage, and tumor clones in the peritoneal fluid were already able to implant in ovarian tissues at that moment.

Our analysis demonstrated that all metastatic samples from this patient were related to cluster P1, not P2, suggesting that the metastatic ability of ancestry clones was more accelerated in cluster P1. Based on this connection, we found that different cancer-related pathways were altered in the early divergent clones (cluster P1 and M vs. cluster P2). JAK/STAT signaling pathway genes including JAK2, known to be related to tumor migration through the epithelial-mesenchymal transition (EMT) [24], were only amplified in clusters P1 and M, supporting the hypothesis that clones in these clusters might be under migration pressure. In contrast, genes involved in cell adhesion pathways were only amplified in cluster P2, indicating that the clones in cluster P2 might be under an opposite pressure to clusters P1 and M.

Whether metastasis requires mutations beyond those required to drive the primary tumor is controversial [25]. In oropharyngeal squamous cell carcinoma, phylogenetic reconstruction according to somatic point mutations showed that metastatic samples arose as a late event [26]. In pancreatic cancer, seeding metastasis may require driver mutations beyond those required for primary tumors, and phylogenetic trees across metastases show organ-specific branches [27]. On the contrary, in HGSC, peritoneal seeding may arise with little accumulation of somatic mutations and copy number alterations. We could not identify the known driver variants causing transcoelomic metastasis in our patient.

In our study based on exome sequencing, all metastatic clones (cluster M) diverged together at a late stage, and the clusters of the primary tumor were distributed in both ovaries (non-spatial clusters). Our results provide a clue that some clones in the primary tumor can have metastatic potential, and that transcoelomic metastasis might be a simple spreading process using existing metastatic ability rather than supporting the previous tumor evolution models (linear [28], parallel [29], or mixed [30]). Regarding the clinical importance of transcoelomic metastasis in HGSC, it is surprising that few additional mutations were found in peritoneal seeding samples. This finding indicates the possibility that the microenvironment, including factors such as stromal cells, might play a role in fostering peritoneal implantation and cancer cell growth by secreting inducing factors [31].

Our study may help to further our understanding of tumor progression during HGSC. The data suggest that clones in peritoneal implants may not be more resistant than primary tumors in some patients. With the increasing clinical use of bioinformatics, developing methods that utilize the large amount of data to categorize patients into prognostic and treatment groups has become increasingly important [32]. This study suggests that patterns of intratumor heterogeneity between primary and metastatic clones might be the key for identifying the most appropriate treatment strategies for patients. In cases with metastatic patterns similar to the patient in this study (e.g., transcoelomic metastasis arising with little genetic alteration accumulation compared with primary tumors), debulking surgery might be useful to achieve optimal cytoreduction through adjuvant chemotherapy. If we identify those groups where seeding metastasis may require driver mutations beyond those required for primary tumorigenesis, debulking surgery might not be useful. In these instances, we should focus instead on the targeted therapy associated with driver mutations in metastatic lesions.

This study may provide important information for those who would like to evaluate tumor evolution in a larger cohort. For future studies evaluating clonal evolution in epithelial ovarian cancer, the following should be considered. First, the presence of mutations identified concurrently in most samples should be validated in a large number of cohorts in order to identify the key regulators in early tumorigenesis. Second, the clonal relationship between various metastatic sites from peritoneal seeding should be evaluated to identify the role of the microenvironment. Further studies are required to document the differences in genomic profiles between various metastatic sites such as the omentum, diaphragm, spleen, and pelvic peritoneum. This approach may elucidate the key regulators in the distinct metastatic characteristics of epithelial ovarian cancer. Third, genomic alterations other than somatic mutations and copy number changes should be considered to identify the unveiled driver variant causing tumor progression. Recently, the microRNA expression profile of an omental metastatic tumor was found to differ from that of the primary tumor in epithelial ovarian cancer, suggesting that microRNA might play role in tumor progression in metastatic tissues [33]. Another group reported that the genomic rearrangement landscapes of metastatic lesions differ from those of primary ovarian cancer [34].

## Conclusion

We performed whole exome sequencing and copy number analysis for multiple primary and metastatic samples within an individual patient. Our research showed that HGSC has diverse intratumor heterogeneity in terms of somatic mutation and copy number variation profiles, but transcoelomic metastasis arises with little accumulation of genetic alterations in this patient.

## Additional file

Additional file 1: Table S1. Summary of analysis platforms for each sample. **Table S2.** Summary of sequencing qualities. **Table S3.** Full list of non-synonymous somatic mutations with annotations. **Table S4.** The sequences of multiplex PCR primers used for validation. **Table S5.** List of functional non-synonymous SNPs in the blood sample. **Table S6.** Comparison of the normalized allele frequency between clusters P1 and P2. **Table S7.** Functional analysis of coding genes within the somatic copy number alteration regions. **Table S8.** Somatic copy number alterations in frequently observed segments in the TCGA project. **Table S9.** Somatic copy number alterations in 22 druggable targets reported in the TCGA project.
Additional file 2: Supplementary Note 1. Criteria for functional prediction algorithms and conservation scores. **Supplementary Note 2.** Urls for analysis programs.
