# Supplementary material for: Tumor evolution and intratumor heterogeneity of an epithelial ovarian cancer investigated using next-generation sequencing
Source: BMC Cancer. 2015 Feb 26;15:85. doi: 10.1186/s12885-015-1077-4 (PMC4346117; doi:10.1186/s12885-015-1077-4)
Supplement: Additional file 2: Supplementary Note 1. — Criteria for functional prediction algorithms and conservation scores. Supplementary Note 2. Urls for analysis programs. [file 12885_2015_1077_MOESM2_ESM.docx]

Supplementary Note 1. Criteria for functional prediction algorithms and conservation scores

| (1) | SIFT: <0.05 (predicted as deleterious) |
| --- | --- |
| (2) | Polyphen-2 HDIV: >0.909 (predicted as probably damaging) |
| (3) | Polyphen-2 HVAR: >0.957 (predicted as probably damaging) |
| (4) | LRT: annotated as “D”(deleterious) |
| (5) | MutationTaster: annotated as “A” (disease causing automatic) or “D” (disease causing) |
| (6) | MutationAssessor: annotated as “high” or “medium” |
| (7) | FATHMM: <-1.5 (predicted as damaging) |
| (8) | GERP++: >3.0 |
| (9) | PhyloP: >1.5 |
| (10) | Siphy: >12 |

The consensus of functional prediction was calculated as the number of algorithms for which the criteria were met (1)-(7) divided by the number of algorithms. The consensus of conservation was calculated as the number of algorithms for which the criteria were met (8)-(10) divided by the number of algorithms. If the consensus of functional prediction was higher than 0.8, and the consensus of conservation was higher than 0.5, we considered the mutation a functional mutation. We also classified a somatic mutation as a functional mutation when the mutation was a stopgain or listed in the COSMIC database.

Supplementary Note 2. Urls for analysis programs

| (1) | Novoalign V2.07.18 http://www.novocraft.com/ |
| --- | --- |
| (2) | Picard v1.67 http://picard.sourceforge.net/ |
| (3) | SeattleSeq Annotator 137 server version 8.07 http://snp.gs.washington.edu/SeattleSeqAnnotation137/index.jsp |
